# Modification of BTLA signaling motifs in TIL exhibits positive signals that mediate anti-tumor control

**DOI:** 10.1186/2051-1426-3-S2-P46

**Published:** 2015-11-04

**Authors:** Krit Ritthipichai, Cara Haymaker, Yared Hailemichael, Minying Zhang, Roza Nurieva, Patrick Hwu, Chantale Bernatchez

**Affiliations:** 1 Department of Melanoma Medical Oncology, The University of Texas M.D. Anderson Cancer Center, Houston, TX, USA; 2 University of Texas Graduate School of Biomedical Sciences, Houston, TX, USA; 3 Department of Immunology, The University of Texas M.D. Anderson Cancer Center, Houston, TX, USA

## Background

Our recent data have shown that a subset of CD8^+^BTLA^+^ was the strongest predictive biomarker of response to adoptive T cell therapy using tumor-infiltrating lymphocytes. BTLA, B-and-T lymphocyte attenuator, is known as an inhibitory molecule in different immune cells. Its cytoplasmic region contains three motifs (Grb2, ITIM, and ITSM). ITIM and ITSM are inhibitory motifs that suppress T cell function upon ligation of the BTLA ligand, HVEM (Herpes virus entry mediator). However, the physiological function of Grb2 motif, known to interact with p85 PI3K, remains unclear. The aim of this study was to investigate the role of the Grb2 motif of BTLA in CD8^+^ TIL and murine CD8^+^ T cells.

## Methods

To dissect the signaling pathway of BTLA's motifs, we generated retroviral vectors containing BTLA and its mutants by substitution of tyrosine (Y) for phenylalanine (F) in either the Grb2 motif (∆Grb2) or ITIM and ITSM (∆ ITIM and ITSM). BTLA and its mutants were overexpressed in either CD8^+^BTLA^-^human TIL or BTLA^-/-^ mouse T cells to uncover BTLA downstream signaling pathways and *in vivo* anti-tumor effect following TIL transfer.

## Results

AKT and MAPK pathway was significantly suppressed in the Grb2 mutant upon HVEM ligation. Greater proliferation was pronounced in ITIM and ITSM mutant mouse T cells when stimulated with DCs pulsed with the cognate peptide, while less proliferation was observed when Grb2 was inactivated. NOD scid gamma (NSG) mice were engrafted human derived melanoma and TIL overexpressing BTLA mutants for the investigation of an anti-tumor effect. ITIM and ITSM mutant TIL exhibited better tumor burden control and were present at higher frequency in peripheral blood following adoptive transfer.

## Conclusions

Our study revealed that the Grb2 motif provided a positive signal that favors anti-tumor responses. Therefore, the strategy to inactivate ITSM and ITSM may enhance persistence following infusion and resulting in mediating tumor control.

